# Histone acetyltransferase PfGCN5 regulates stress responsive and artemisinin resistance related genes in *Plasmodium falciparum*

**DOI:** 10.1038/s41598-020-79539-w

**Published:** 2021-01-13

**Authors:** Mukul Rawat, Abhishek Kanyal, Aishwarya Sahasrabudhe, Shruthi Sridhar Vembar, Jose-Juan Lopez-Rubio, Krishanpal Karmodiya

**Affiliations:** 1 Department of Biology, Indian Institute of Science Education and Research Pune, Dr. Homi Bhabha Road, Pashan, Pune, 411 008 India; 2 Institute for Bioinformatics and Applied Biotechnology, Bengaluru, Karnataka India; 3 Laboratory of Pathogen-Host Interactions (LPHI), UMR5235, CNRS, INSERM, Montpellier University, Montpellier, France

**Keywords:** Parasite genomics, Chromatin remodelling, Histone post-translational modifications

## Abstract

*Plasmodium falciparum* has evolved resistance to almost all front-line drugs including artemisinin, which threatens malaria control and elimination strategies. Oxidative stress and protein damage responses have emerged as key players in the generation of artemisinin resistance. In this study, we show that PfGCN5, a histone acetyltransferase, binds to the stress-responsive genes in a poised state and regulates their expression under stress conditions. Furthermore, we show that upon artemisinin exposure, genome-wide binding sites for PfGCN5 are increased and it is directly associated with the genes implicated in artemisinin resistance generation like BiP and TRiC chaperone. Interestingly, expression of genes bound by PfGCN5 was found to be upregulated during stress conditions. Moreover, inhibition of PfGCN5 in artemisinin-resistant parasites increases the sensitivity of the parasites to artemisinin treatment indicating its role in drug resistance generation. Together, these findings elucidate the role of PfGCN5 as a global chromatin regulator of stress-responses with a potential role in modulating artemisinin drug resistance and identify PfGCN5 as an important target against artemisinin-resistant parasites.

## Introduction

Malaria is a life threatening infectious disease caused by parasites from the genus *Plasmodium,* with an estimated 228 million cases worldwide in year 2018^1^. The *Anopheles* mosquito serves as a vector for varied species of the human malaria parasite namely *P. falciparum, P. vivax, P. ovale, P. malariae* and *P. knowlesi*. Of these five species, *P. falciparum* causes the most lethal form of malaria. Since *Plasmodium* completes its life cycle in two different hosts, it requires mechanisms for coordinated modulation of gene expression^2^. An efficient transcriptional and post-transcriptional regulation of gene expression enables it to establish chronic infection in humans^3,4^. Moreover, recent studies have attested the importance of epigenetic mechanisms in regulation of gene expression^3,5–9^. Morphological changes observed during the development of the malaria parasite in erythrocytes are also governed by the fine-tuning of gene expression^2^. Most of the genes in *Plasmodium* are reported to be poised (genes exhibit high levels of histone activation marks but no transcription), which favors the plasticity of its gene expression programs^5,10,11^.

During the asexual life cycle, when *Plasmodium* is developing within mature red blood cells (RBCs), it is exposed to different kinds of environmental and physiological stresses. For instance, during the trophozoite stage the parasite converts haemoglobin to hemozoin, leading to the accumulation of reactive oxygen species (ROS) and consequently oxidative stress^12–14^. Another characteristic of malarial infection is the acute cyclical episodes of fever, with an increase in temperature to 41 °C for about 2–6 h. This periodic febrile response is triggered by the release of merozoites from RBCs^15–17^. Since *Plasmodium* faces these stress conditions during each of its infectious cycles, it has possibly evolved mechanisms to resist the metabolic perturbations caused thereby. Recent studies also suggest role of stress responsive genes in drug-resistance against antimalarial drugs^18,19^. Several classical antimalarial drugs like chloroquine^20^, sulfadoxine-pyrimethamine^21^ and mefloquine^22^ are no longer effective against *P. falciparum*^23,24^. Currently, artemisinin based combination therapy is considered as the last line of defense against *P. falciparum* malaria^24^. However, since 2009, alarming reports of resistance against artemisinin have emerged in Southeast Asia. This region has historically served as the epicenter for emergence of anti-malarial drug resistance^25–27^. Recent reports from Eastern India have also suggested the presence of artemisinin resistant parasites based on pharmacokinetic and genetic parameters like increased parasite clearance half-life and novel Kelch13 mutations^28,29^.

Currently, there is limited knowledge on the mechanism by which the parasites develop resistance against artemisinin. Artemisinin resistant parasites are characterized by longer ring stage and shorted trophozoite/schizont stage and reduced drug susceptibility at the ring stage of asexual growth^19^. Multiple transcriptomics studies have revealed dormancy, oxidative stress response and protein metabolism to be associated with the artemisinin drug resistance generation^18,19,26,30–33^. Another mechanism recently identified to play an important role in artemisinin resistance is the delayed haemoglobin endocytosis and digestion^34–36^. Kelch13 along with other proteins comprise this endocytic compartment which mediate haemoglobin uptake. Inactivation of several of the proteins of the Kelch13-defined compartment renders artemisinin resistance by delayed endocytosis of haemoglobin^34^. Biochemical studies have shown decreased levels of Kelch13 in the resistant parasites possibly due to the reduced stability of mutant protein^35^. Furthermore, peptidomics analysis showed lower abundance of peptides derived from haemoglobin digestion in the artemisinin-resistant parasites indicating either reduced endocytosis or dysregulated digestion of the haemoglobin.

Unfortunately, the global transcriptional regulators of stress responses and drug resistance generation remain unexplored in *P. falciparum*. Previous studies in higher (e.g., humans) and lower (e.g., *Toxoplasma gondii*) eukaryotes demonstrated that GCN5, a histone acetyltransferase plays an important role during stress conditions, where it has been associated with high level of transcriptional reprogramming required for stress adaptation^37–41^. GCN5 is conserved in *Plasmodium* species and till date, only two subunits of the GCN5 complex, namely PfGCN5 and PfADA2 are identified^42,43^. PfGCN5 has a long N-terminal tail with no identifiable domains that is absent in higher eukaryotes and yeast two hybrid studies have suggested that this N-terminal tail might be playing an important role in protein–protein interactions^44^. Multiple transcription initiation sites were identified for PfGCN5 in the recently published comprehensive atlas of transcription initiation events in *P. falciparum* asexual stages at single nucleotide-resolution^45^. *In-vitro* studies have suggested that recombinant PfGCN5 can acetylate K9 and K14 residues of histone H3^42^. Previous studies using DNA microarray have suggested that there is a weak but positive correlation between PfGCN5 and H3K9ac mark^46^. Recently Bhowmick et al. showed that proteolytic cleavage of PfGCN5 is necessary for its function^47^. PfGCN5 was found to undergo cysteine protease cleavage into several cleaved products and the proteolytic processing is important for its nuclear acetylation function^47^.

In this study, we dissected the role of PfGCN5 under various physiological stress conditions in *P. falciparum* during intraerythrocytic development cycles. With the help of chromatin immunoprecipitation coupled high-throughput sequencing (ChIP-seq) and transcriptomic (RNA-sequencing) analyses, we show that PfGCN5 activates genes that are important for the maintenance of parasite cellular homeostasis during various stress conditions. Collectively, our data identify histone acetyltransferase, PfGCN5 as a key chromatin regulator of stress responsive genes and reveals its important role in emergence of artemisinin drug resistance.

## Results

### PfGCN5 is associated with stress responsive genes

**Figure 1:**
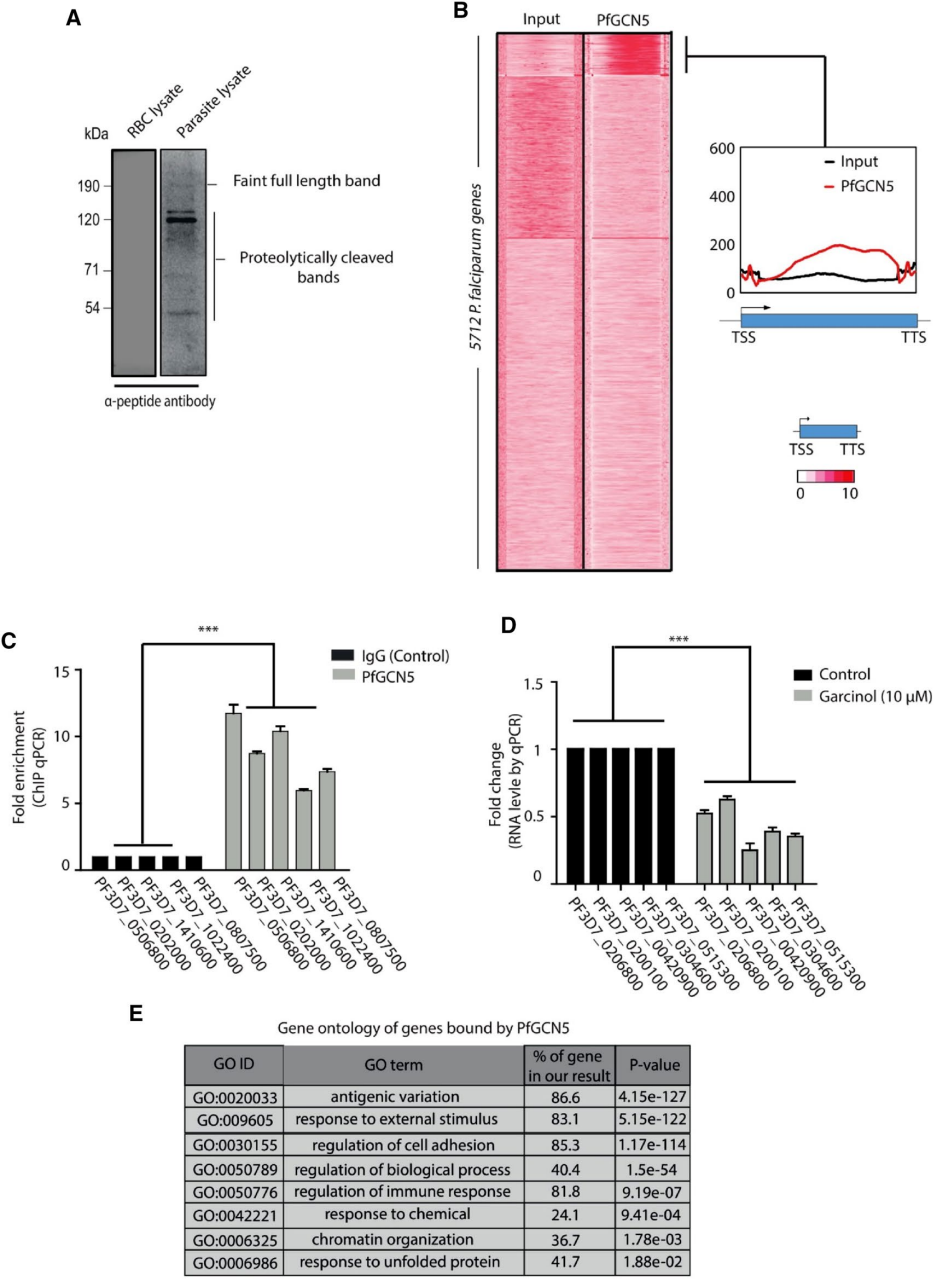
PfGCN5 is associated with stress responsive genes. (**A**) An anti-PfGCN5 antibody was generated using the N-terminal peptide of the PfGCN5. Specificity of the antibody was tested using parasite protein lysate from asynchronous culture and uninfected RBCs. Western blotting results indicated presence of more than one form of PfGCN5 along with the full-length protein*.* The lower size bands are products of proteolytic cleavage as reported earlier^47^ and explained in Supplementary Figure S1B. (**B**) Heat map showing the ChIP-seq tag counts at 5712 *P. falciparum* genes for PfGCN5. PfGCN5 was found to be enriched mostly at the 3′ end of the genes and towards the centre of the genes. (**C**) ChIP-qPCR of selected genes confirms PfGCN5 binding to ChIP-seq targets. The results are shown as fold enrichment of ChIP performed with PfGCN5 α-peptide antibody versus non-immune IgG. Significance was determined using a paired t-test. *p < 0.05; ***p < 0.005. (**D**) RT-qPCR of the gene bound by PfGCN5 in the presence and absence of garcinol (10 µM). Significance was determined using a paired t-test. *p < 0.05; ***p < 0.005. (**E**) Gene ontology analysis of the PfGCN5 bound gene identified using ChIP sequencing. Stress responsive genes were overrepresented in gene ontology analysis.

While paralogs of GCN5 are well studied in multiple systems, little is known about the function of GCN5 in *P. falciparum*. PfGCN5, encoded by *PF3D7_0823300,* contains histone acetyltransferase (HAT) and bromo (for binding to acetylated histones) domains at its C-terminal end (Supplementary Figure S1A)^42,43^. To gain further insight into the function of PfGCN5 during asexual growth, we generated polyclonal antibodies against a specific peptide from the N-terminal region of PfGCN5 (amino acids 9–25; called α-peptide antibody; Supplementary Figure S1A). As expected, we detected multiple bands of PfGCN5 (190, 124, 120, 70 kDa and 43 kDa bands) (Fig. 1A). PfGCN5 is known to have multiple proteolytic products as described earlier^47^. However, the differences in fragment pattern between the two antibodies can be attributed to the fact that the previous study has used an antiserum recognizing the C-terminal region of the PfGCN5 as compared to the N-terminal PfGCN5 antibody generated in this study. To corroborate the differential banding patterns, we performed an in silico proteolytic analysis using PROSPER (https://prosper.erc.monash.edu.au/queue.pl) and confirmed proteolytically cleaved fragments similar to one identified in our Western blotting. Differential profile observed between two different PfGCN5 antibodies is explained through schematic shown in Supplementary Figure S1B. Further to confirm the specificity of α-peptide PfGCN5 antibody, we performed immunoprecipitation followed by mass spectrometry. PfGCN5 was identified in the mass spectrometry analysis (4^th^ protein in the list) whereas it was absent in the IgG immunoprecipitation (Supplementary Table S1). We also identified PfADA2, a known interacting partner of PfGCN5 in our immunoprecipitation confirming that the antibody raised is specific to PfGCN5.

Next, to comprehend the transcriptional regulation mechanisms of PfGCN5, we performed chromatin immunoprecipitation coupled high-throughput sequencing (ChIP-seq) using α-peptide PfGCN5 antibodies. ChIP-seq was performed at an early trophozoite stage (24 hpi) of parasite growth as PfGCN5 exhibits high mRNA expression at this stage (Supplementary Figure S1C)^48^. Peaks of local enrichment of PfGCN5 were determined after sequence alignment and normalization to input sequences using the MACS2 peak calling software. In total, we identified 782 high confidence binding sites (fold enrichment >  = 2; q value < 0.1) with α-peptide PfGCN5 antibody, which corresponds to 760 genes.

We measured PfGCN5 density distribution (as measured by α-peptide antibody) across all 5712 *P. falciparum* genes. PfGCN5 was found to be enriched at the 3′ end and centre of the gene body of target genes identified by MACS2 analysis (Fig. 1B). Further, to validate the peaks obtained in ChIP-seq, we performed ChIP-qPCR on randomly selected genomic loci enriched for PfGCN5 and confirmed its binding (Fig. 1C). Further to functionally validate whether PfGCN5 binding regulates the expression of genes identified in ChIP sequencing, we performed chemical inhibition of PfGCN5 using Garcinol inhibitor. It is an inhibitor of recombinant PfGCN5 and shows an IC_50_ of ~ 15 μM against 3D7 strain^49^ (Supplementary Figure S1D,E). We performed quantitative real-time PCR (RT-qPCR) to check the expression level of few genes in presence and absence of garcinol (10 µM). Interestingly, in the presence of garcinol there was a decrease in the transcript expression level of genes bound by PfGCN5 (Fig. 1D) indicating their possible regulation by PfGCN5. Lastly, gene ontology (GO) analysis of PfGCN5-bound genes indicated pathways like “chromatin organization”, “antigenic variation”, “regulation of immune response”, and “response to unfolded proteins” (Fig. 1E). Altogether this suggests that PfGCN5 play an important role in the regulation of stress responsive and stimuli-dependent genes in *P. falciparum*.

### PfGCN5 is not a general transcription coactivator; it is specifically associated with stress/stimuli associated genes

**Figure 2:**
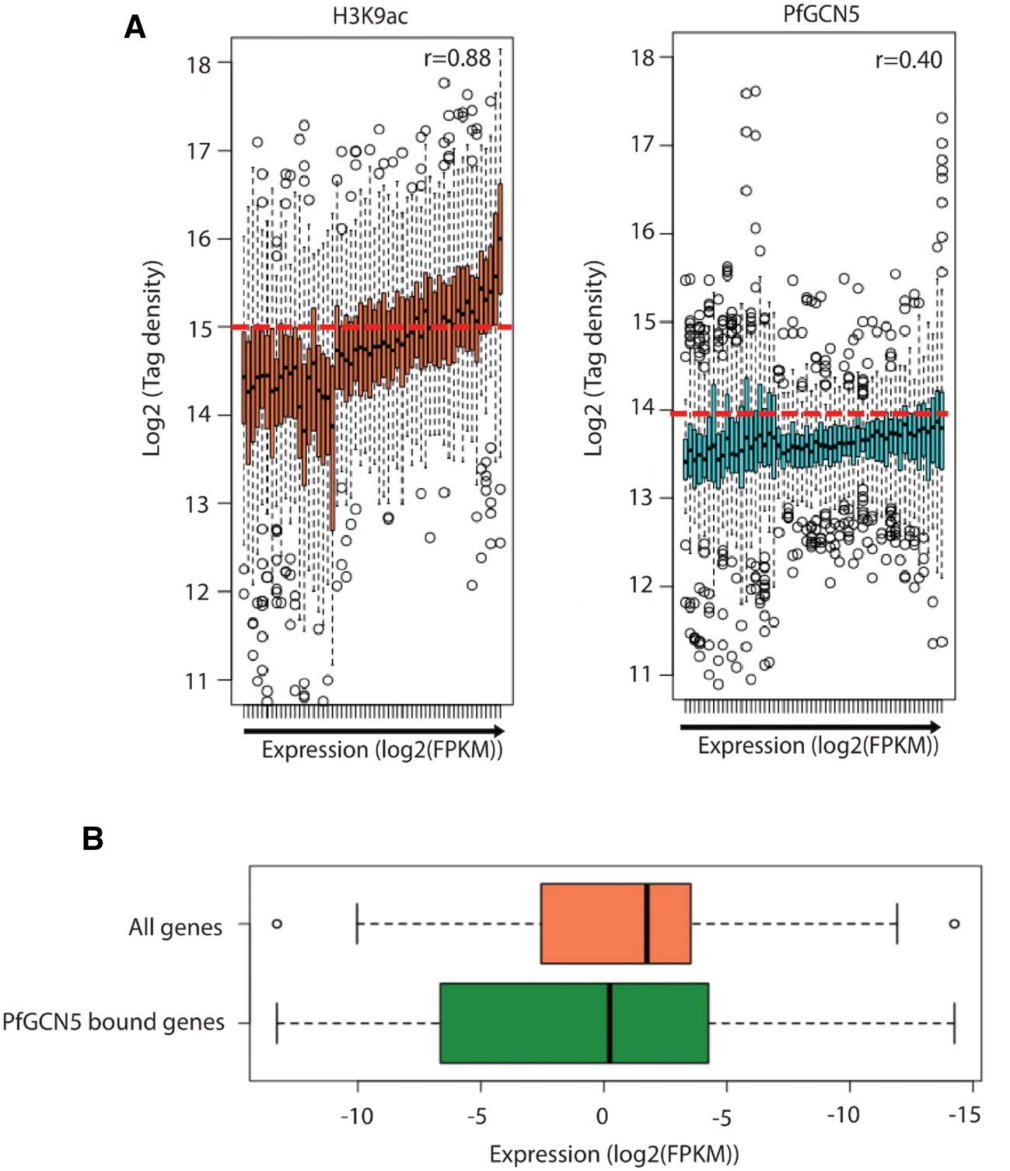
PfGCN5 is not a general transcription coactivator; it is specifically associated with stimuli associated genes. (**A**) Box and whisker plots representing the correlation of genome-wide H3K9ac prevalence and PfGCN5 occupancy with the global gene expression. Absence of global correlation was found for the recruitment of PfGCN5 and gene expression. This indicates that PfGCN5 is not a general transcription coactivator. (**B**) The expression level of the genes bound by PfGCN5 in comparison to all the genes in *P. falciparum* is represented by the box plots. PfGCN5 is associated with highly as well as least expressed genes.

Next, we investigated how PfGCN5 binding relates to transcriptional activity of a gene at the trophozoite stage. We systematically calculated the enrichment levels of PfGCN5 and H3K9ac^5^, a general activation mark, at the gene body of all *P. falciparum* genes and compared it to the relative expression levels of genes as evaluated by RNA-sequencing analysis during trophozoite stage of 3D7 parasites. As expected, we observed a positive correlation between H3K9ac enrichment and the expression status of the downstream gene (Fig. 2A; left panel). On the other hand, we observed no/little correlation between PfGCN5 gene-body occupancy and the expression of nearby genes (Fig. 2A; right panel). Genes with either high or low expression levels (outlier points for log2 read density) showed high PfGCN5 occupancy (Fig. 2A), suggesting that PfGCN5 binds to both active and suppressed/poised genes. In order to confirm this, we compared the expression levels of genes bound by PfGCN5 and contrasted them with the expression of all the *P. falciparum* genes. The expression level of PfGCN5 bound genes spreads from high expression to low expression values (Fig. 2B) indicating its presence on expressed as well as suppressed genes. Thus, absence of global correlation with transcription and occupancy on suppressed/poised as well as active genes, suggest that PfGCN5 is not a general transcriptional co-activator rather it may specifically regulate stress responsive genes in *P. falciparum*.

### PfGCN5 is a specific regulator of stress responsive genes

**Figure 3:**
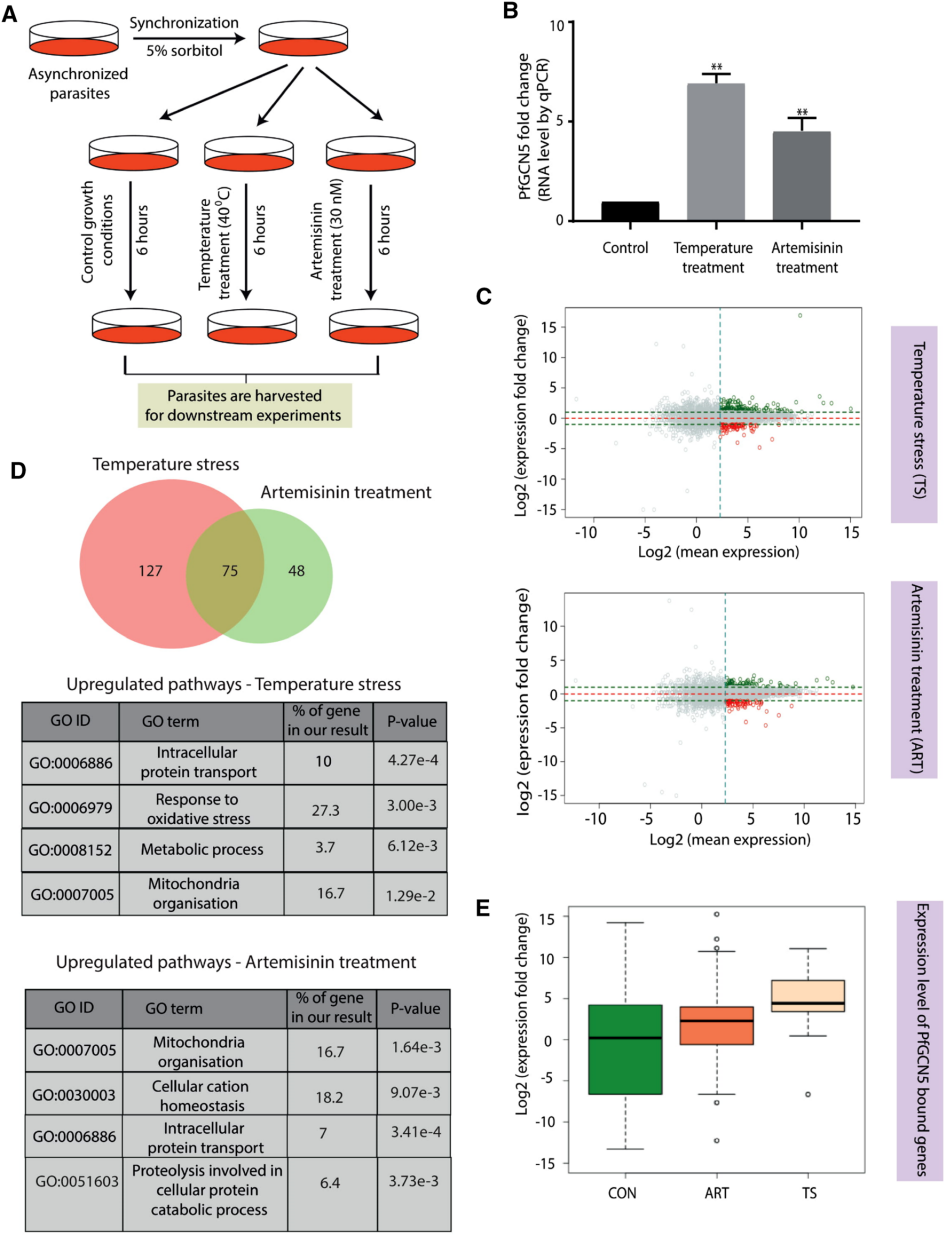
PfGCN5 is a specific stress regulator. (**A**) Schematic representation of the pipeline used for the stress experiment. (**B**) Change in the expression level of PfGCN5 during various stress conditions (N = 3). PfGCN5 is found to be upregulated during heat stress and artemisinin treatment conditions. Significance was determined using a paired t-test. *p < 0.05; ***p < 0.005. Data shows the mean ± SEM for three independent experiments. (**C**) MA plot showing the deregulation in the expression of protein coding genes during artemisinin treatment with 30 nM concentration for 6 h and during temperature stress at 40 °C for 6 h. (**D**) Venn diagram showing the genes upregulated during temperature stress and artemisinin treatment. Table representing the pathways enriched for the genes upregulated during temperature stress and artemisinin treatment. Gene ontology was performed using PlasmoDB. (**E**) Expression profiles of the genes bound to PfGCN5 during stress conditions. PfGCN5 bound genes are upregulated upon stress induction as compared to the control condition.

Next, we decided to look into the role of PfGCN5 during stress conditions. Tightly synchronized ring stage parasites (17 hpi) were exposed to two different physiological stress conditions; temperature (40 °C) and artemisinin drug (30 nM) exposure for 6 h (Fig. 3A). We have used a temperature which is widely used in the field that does not results in stalling of parasite growth^15,16^. Concentration of the artemisinin used for our experiments was also very low (30 nM) for 6 h in comparison to physiological concentration used for RSA experiments (700 nM) to avoid any stalling of parasite growth. We have also monitored the growth of the parasites using giemsa stain upon heat stress and artemisinin treatment and did not observe any change in the parasitemia or stalling of growth post treatment (data not shown). In order to confirm the stress response, we looked at the expression level of genes, which are known to be upregulated during stress conditions in *P. falciparum*. For temperature stress we looked at the expression level of the heat shock protein, HSP70 (Supplementary Figure S2A)^16,50^. Since the artemisinin is known to induce oxidative stress through production of reactive oxygen species, we confirmed the oxidative stress by validating the expression levels of glutathione S-transferase and superoxide dismutase (Supplementary Figure S2A)^13,51^. PfGCN5 was found to be upregulated several folds upon physiological stress conditions as shown by RT-qPCR using gene specific primers (Fig. 3B). To identify the genes that are deregulated under these stress conditions, we performed transcriptomic analysis using RNA-sequencing and identified 123 and 202 genes (> twofold change and minimum 5 FPKM value) deregulated upon artemisinin and high temperature exposure, respectively (Fig. 3C and Supplementary Table S2). Genes showing deregulation during stress conditions were also validated by RT-qPCR (Supplementary Figure S2B). Most of the upregulated genes during artemisinin and temperature stress conditions are reported to maintain cellular homeostasis during stress conditions by regulating different pathways. Pathways related to “response to oxidative stress”, “protein transport” and “mitochondria organisation” were enriched under temperature stress condition (Fig. 3D). During artemisinin treatment, pathway related to “cellular cation homeostasis” was enriched along with “proteolysis involved in cellular protein catabolic process”, “protein transport” and “mitochondria organisation”. To further dissect the functional correlation between transcriptome deregulation and recruitment of PfGCN5 under different stress conditions, we performed ChIP-sequencing for PfGCN5 using α-peptide antibody during both temperature and artemisinin stress conditions. Notably, most of the PfGCN5 bound genes are upregulated under artemisinin and temperature stress conditions (Fig. 3E), indicating that PfGCN5 is possibly associated with the activation of stress responsive genes.

### PfGCN5 helps in maintenance of homeostasis during artemisinin treatment

**Figure 4:**
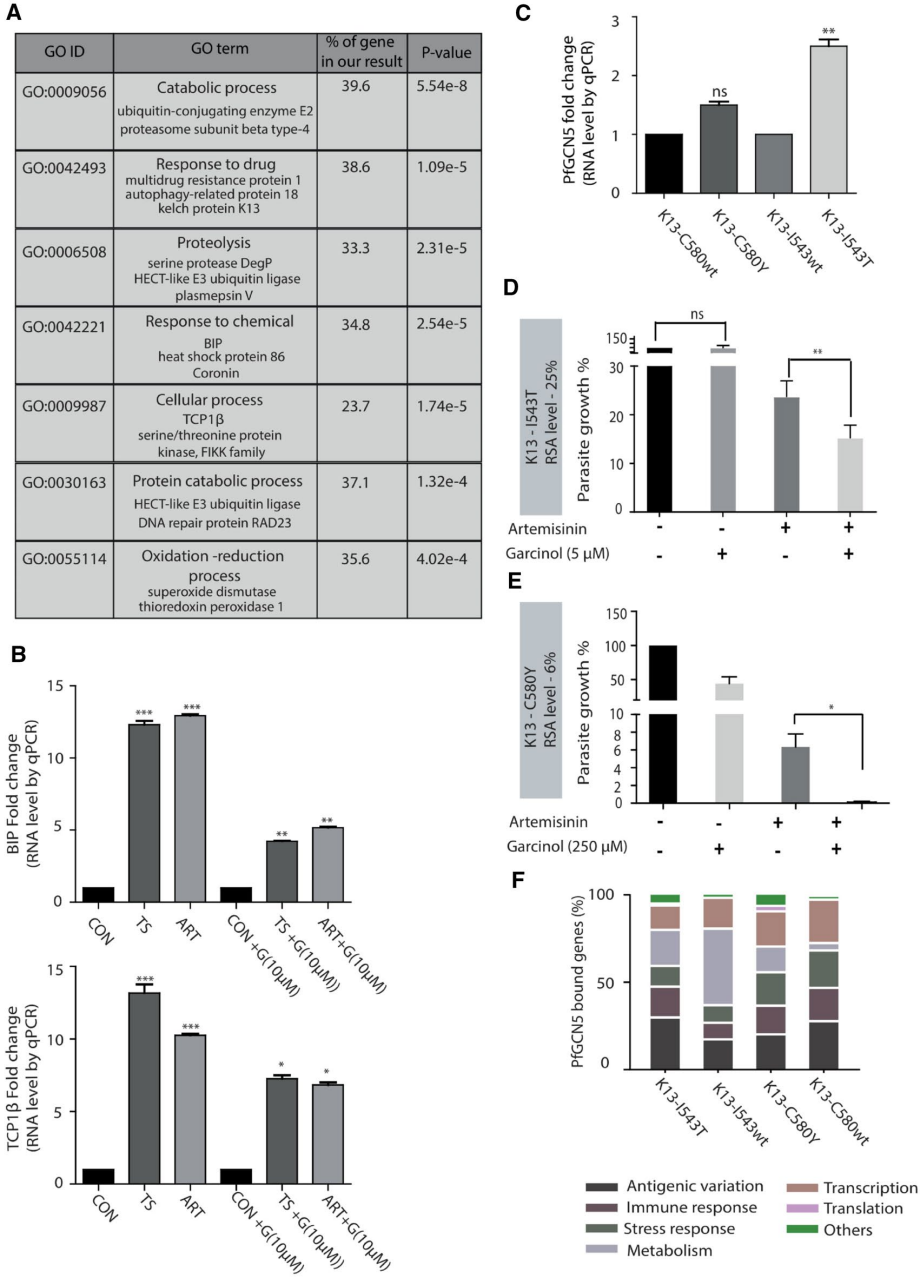
PfGCN5 shows prolific genomic binding during artemisinin treatment and in artemisinin resistant parasites. (**A**) Gene ontology analysis of the genes which are exclusively bound by PfGCN5 during artemisinin treatment. PfGCN5 is found to be enriched on the genes which are known to be deregulated in artemisinin resistant parasites indicating the possible role of PfGCN5 during resistance generation. (**B**) RT-qPCR results show upregulation of BiP and TCP1β during stress conditions. Garcinol treatment (10 µM) leads to decreased expression of BiP and TCP1β during stress conditions. Data shows the mean ± SEM for three independent experiments. Significance was determined using a paired t-test. *p < 0.05; ***p < 0.005. (**C**) Transcript level of expression of PfGCN5 in artemisinin resistant strains, K13-I543T (MRA-1241) and K13-C580Y (MRA-1236) in comparison to their sensitive counterparts, K13-I543wt (MRA-1253) and K13-C580wt (MRA-1254), respectively. (**D**,**E**) Change in the percentage parasite survival estimated through Ring Survival Assay (RSA) in presence of PfGCN5 inhibitor garcinol. (**D**) K13-I543T (MRA-1241) parasites were treated with 5 µM garcinol and (**E**) K13-C580Y (MRA-1236) parasites were treated with 250 µM garcinol. Presence of garcinol decreases the artemisinin resistance in K13-I543T (MRA-1241) at a concentration which has otherwise no effect on parasite growth. Higher concentration of garcinol was used with a significant decrease in resistance level in K13-C580Y (MRA-1236) parasites. (**F**) Gene ontology analysis of the genes which are bound by PfGCN5 in K13-I543T (MRA-1241), K13-C580Y (MRA-1236), K13-I543wt (MRA-1253), K13-C580wt (MRA-1254).

Responses to oxidative stress and protein damage are shown to mediate emergence of artemisinin resistance in malaria parasites^18,19,31,52^. Interestingly, 775 new PfGCN5 bound sites were acquired under artemisinin stress condition as indicated by ChIP-sequencing of PfGCN5. Moreover, gene ontology of newly acquired PfGCN5 bound genes under artemisinin stress conditions includes pathways such as “catabolic process’, “response of chemical”, “oxidation–reduction process” and “response to drug”, which are known to be deregulated in artemisinin resistant parasites (Fig. 4A). Importantly, several genes known to play an important role in the mechanism of artemisinin resistance are bound by PfGCN5 under artemisinin treatment (Fig. 4A). Of interest is the binding of PfGCN5 at BiP and T-complex protein 1 (TCP1) ring (TRiC) chaperone genes. It is plausible that the higher expression of PfGCN5 upregulates BiP and TRiC chaperones, thus assisting the unfolded protein response in artemisinin resistant parasites^52^. Furthermore, to understand the role of PfGCN5 in artemisinin drug resistance, we performed RT-qPCR during stress conditions both in presence and absence of garcinol (10 µM). We found that both BiP and TCP1 β (T complex protein 1 subunit beta) were upregulated during the stress conditions (Fig. 4B). However, upon garcinol treatment, there was a marked decrease in the expression level of BiP and TCP1 β under stress conditions (Fig. 4B). A previous study investigating the differential proteome under artemisinin treatment (35 nM) identifies 46 proteins to be upregulated^53^. We found that out of these 46 proteins, 21 were bound by PfGCN5 under artemisinin treatment in our study (Supplementary Figure S3).

Further to dissect the role of PfGCN5 in artemisinin drug resistance emergence and maintenance, we looked at the transcript level of PfGCN5 in artemisinin resistant lines. PfGCN5 was upregulated in artemisinin resistant line K13-I543T (MRA-1241, RSA ~ 25%) by 2.5-fold than its sensitive counterpart during early trophozoite stage of the parasite (Fig. 4C). Another artemisinin resistant line, K13-C580Y (MRA-1236, RSA ~ 6%) had 1.5-fold upregulation in PfGCN5 than its sensitive counterpart during early trophozoite stage of the parasite (Fig. 4C). We wondered if the inhibition of PfGCN5 activity resulted in change in drug sensitivity of the artemisinin resistant lines: K13-I543T and K13-C580Y. Ring survival assay (RSA) was performed in absence and presence of 5 µM garcinol which has minimal effect on normal parasite growth as indicated by the growth of parasite in the bar graph (Fig. 4D). We observed a 36.4% decrease in the level of resistance for K13-I543T artemisinin resistant line in the presence of PfGCN5 inhibitor, garcinol (Fig. 4D). Interestingly, garcinol treatment of the artemisinin resistant parasites at higher concentration (close to the IC50 of K13-C580Y at 250 µM); K13-C580Y completely reverses the artemisinin resistance (Fig. 4E) indicating that PfGCN5 plays an important role in artemisinin resistance.

In order to further understand the role of PfGCN5 in artemisinin resistance, we performed PfGCN5 ChIP sequencing in artemisinin resistant K13-I543T (MRA-1241) and K13-C580Y (MRA-1236) and their artemisinin sensitive counterpart K13-I543wt (MRA-1253) and K13-C580wt (MRA1254) using α-peptide PfGCN5 antibody. We investigated the strain specific genes enriched for PfGCN5 binding and called their associated biological processes through the gene ontology analysis. Several biological processes were found to be conserved between the artemisinin sensitive and resistant strain. These primarily include “cellular adhesion”, “response to stimulus” and “antigenic variation”, highlighting their regulation by PfGCN5 across strains (Fig. 4F). Interestingly, a set of genes were uniquely enriched for PfGCN5 occupancy in the resistant strains. While, PfGCN5 was enriched on cellular metabolism and protein translation associated genes in K13-I543T strain, in K13-C580Y it was enriched on genes involved in vesicle fusion, and morphogenesis (Fig. 4F). Deregulation of these biological pathways has been shown to be crucial for artemisinin resistance acquisition in the field isolates of *P. falciparum*^54^. Thus, our findings also reiterate an important aspect of resistance emergence posited earlier, that it is highly dynamic and can be shaped by independent underlying genetic and external environmental factors^18^. Together, these results suggest that PfGCN5 plays an important role in the regulation of stress responses, which are associated with drug resistance emergence.

## Discussion

*Plasmodium* must have evolved efficient machineries to overcome changes in environmental conditions experienced in two different hosts. The ability of *Plasmodium* to develop resistance against artemisinin is attributed to the competent stress responsive pathways and the unfolded protein response machinery, which are activated upon artemisinin exposure^18,19^. Here, we establish the role of the histone acetyltransferase PfGCN5 as a global regulator of stress responsive pathways in *P. falciparum*. Genome-wide analysis of PfGCN5 occupancy shows that it is associated with stress responsive and stimuli dependent genes. Interestingly, PfGCN5 occupancy at various genomic loci was found to establish a transcriptionally poised state, which may allow these genes to be switched on or off immediately in response to stimuli. Such regulation is crucial for the genes implicated in stress response and host immune evasion. We and others have previously shown that H3K14ac, another histone modification mediated by GCN5, is specifically present on poised stress responsive genes in higher eukaryotic systems^55,56^ indicating a conserved role of GCN5 in *P. falciparum*. Together, these results suggest that PfGCN5 is not a general transcription coactivator and it specifically regulates the stress responsive genes in *P. falciparum*.

**Figure 5:**
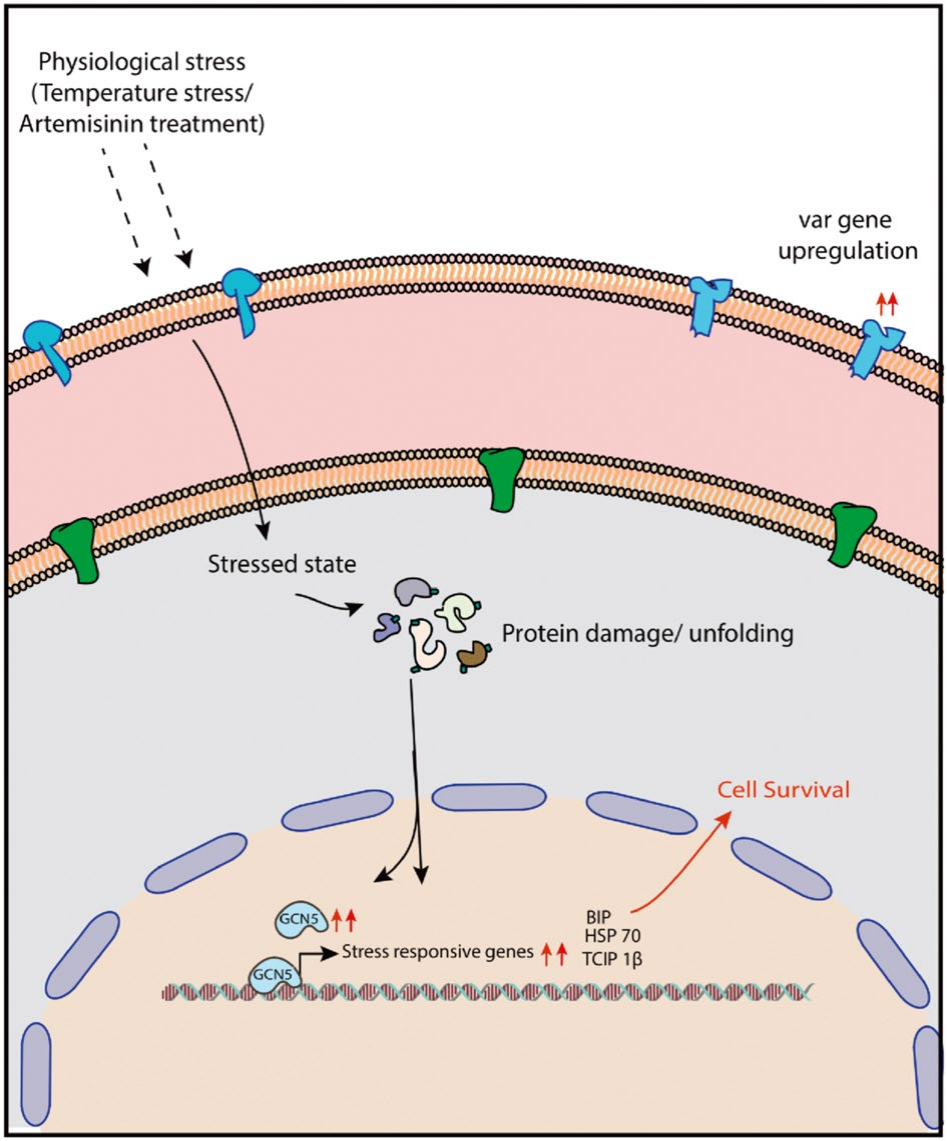
Mechanisms proposed for artemisinin resistance in *P. falciparum*. Model showing the role PfGCN5 in artemisinin resistance generation. Artemisinin treatment leads to random alkylation of proteins, which in turn are ubiquitinated by the protein ubiquitination and subjected to degradation by proteasome degradation. Massive alkylation by artemisinin exposure and/or oxidative stress activates PfGCN5 in the nucleus, which in turn upregulates the stress-responsive and unfolded protein response pathways that help in artemisinin resistance generation.

In order to get insights into the role of PfGCN5, we looked at the level of PfGCN5 transcript as well as genome wide binding sites during stress conditions i.e. heat stress and artemisinin exposure. We found that PfGCN5 is upregulated during stress conditions and its transcript level is comparable to artemisinin resistant parasites. Surprisingly, upon artemisinin treatment PfGCN5 is enriched on the genes important for the development of resistance against artemisinin. Corroborating PfGCN5 genome-wide binding with transcriptome data clearly indicates that PfGCN5 is associated with the genes which are upregulated during stress conditions (e.g. artemisinin exposure). Furthermore, upon interfering with the activity of PfGCN5 using its specific inhibitor, garcinol, we found a significant decrease in the level of artemisinin resistance in the K13-I543T mutant (MR4-1241, RSA-25%) and K13-C580Y (MRA-1236, RSA-6%). This in turn suggests that PfGCN5 is a global regulator of stress responsive genes, and plays an important role in artemisinin resistance maintenance. Moreover, PfGCN5 also regulates transcription regulation of BiP and T complex protein 1 beta subunit beta under stress conditions. Reports from higher eukaryotic systems have suggested that acetylation of BiP results in its dissociation from the protein kinase RNA-like endoplasmic reticulum kinase (PERK), which further results in phosphorylation of eIF2α leading to translation repression^57^. Moreover, we also found PfGCN5 to be enriched at the promoter of the Kelch13 gene, which possibly hints at its transcriptional regulation. Together, these suggest that PfGCN5 may play an important role in drug resistance generation either by directly regulating the expression of the genes important for emergence/maintenance of artemisinin resistance and/or by interacting with various key stress-regulators involved in resistance generation in *P. falciparum* (Fig. 5)*.*

Emergence of drug resistance against artemisinin is one of the biggest hurdles in malaria control and eradication. Recent reports have implicated stress responsive pathways in drug resistance generation (Fig. 5). Understanding the regulation of stress responses is crucial to fathom the pathogenesis of the parasites. Our study identifies PfGCN5 as a global regulator of transcription of stress responsive genes in *P. falciparum*. The outcome of this study could potentially be used to develop and screen inhibitors against drug resistant malaria parasites, which is one of the most prevalent parasitic diseases in the world.

## Experimental procedures

### Parasite culture

*P. falciparum* strain 3D7 was cultured as previously described^58^. Briefly, parasites were cultured in RPMI1640 medium supplemented with 25 mM HEPES, 0.5% AlbuMAX I, 1.77 mM sodium bicarbonate, 100 μM hypoxanthine and 12.5 μg ml^−1^ gentamicin sulfate at 37 °C. Parasites were sub-cultured after every two days. Subculturing was done by splitting the flask into multiple flasks in order to maintain parasitemia around 5%. Hematocrit was maintained to 1–1.5% by adding freshly washed O^+ve^ human RBC isolated from healthy human donors. Synchronization was done with the help of 5% sorbitol in the ring stage. Late stage synchronization was performed using the Percoll density gradient method (63%). Parasitemia was monitored using Giemsa staining of thin blood smear.

### Antibodies

For generating PfGCN5 peptide antibody, online software LBtope was used for selecting the antigenic peptide. PfGCN5 peptide (CEYCNVLYDGNELLRKRK) used for raising antibody was obtained from Apeptide Co., Ltd., China. PfGCN5 peptide was conjugated to Keyhole limpet hemocyanin (KLH) carrier protein for the immunization purpose. Anti-GCN5 peptide antibodies were raised at The National Facility for Gene Function in Health and Disease, IISER Pune. The New Zealand White rabbits (3–4 months old) were used for antibody generation. Antibody were further purified using affinity chromatography on the sulpholink resin.

### Western blotting

Parasites were harvested using 0.15% saponin. Parasites pellets were washed using phosphate buffer saline (PBS). Parasites were lysed using ice cold parasite lysis buffer (TRIS pH 8.0, 150 mM sodium chloride (NaCl), 0.5% nonyl phenoxypolyethoxylethanol (NP-40), 0.5% sodium deoxycholate, 0.1 mM ethylenediaminetetraacetic acid, 1.5 mM magnesium chloride (MgCl_2_), 1X protease inhibitor cocktail (PIC), 1 mM phenylmethylsulfonyl fluoride (PMSF). Three freeze thaw cycles were performed using liquid nitrogen to achieve proper lysis of the parasites. To get rid of debris, parasites were spun at 17,949×*g* for 30 min. Supernatant was transferred to another tube. The lysate proteins were separated on 7.5–12% polyacrylamide gels and transferred to PVDF membrane. The membrane was blocked using 5% skimmed milk and probed using primary antibody overnight at 4 °C. After overnight incubation membranes were washed using 1X Tris-buffered saline, 0.1% Tween 20 (TBST) followed by 1 hr incubation with secondary antibody in TBST (1:5000, Biorad). Three washes were given for 10 min each after the secondary antibody incubation. Blots were developed using Clarity Western ECL substrate (Biorad).

### Mass spectrometry

In order to harvest the parasites, infected RBCs were lysed using 0.15% saponin at 370 °C. Harvested parasites were then lysed using ice cold parasite lysis buffer (20 mM TRIS pH 8.0, 150 mM NaCl, 0.5% NP-40, 0.5% sodium deoxycholate, 0.1 mM EDTA, 1.5 mM MgCl2, 1X PIC, 1 mM PMSF). Lysed parasites were then centrifuged at 20,817×*g* for 30 min at 40 °C. Pre-clearing was performed using recombinant protein G conjugated sepharose beads for 1 h at 40 °C. Precleared lysate was then used for overnight incubation with antibody at 40C. After the overnight incubation of lysate with antibody, sepharose Protein G beads were added to the lysate for 4 h incubation. Washes were done using immunoprecipitation buffer (25 mM TRIS pH 7.9, 5 mM MgCl2, 10% glycerol, 100 mM KCl, 0.1% NP-40, 0.3 mM DTT) followed by elution of the proteins using glycine (pH—2.5). Eluted proteins were neutralized using 1 M Tris pH 8.8. For mass spectrometry analysis samples were digested with trypsin for 16 h at 370 °C. The digested samples were cleared using C18 silica cartridge. Peptides were then analysed using EASY-nLC 1000 system (Thermo Fisher Scientific) coupled to QExactive mass spectrometer (Thermo Fisher Scientific) equipped with nanoelectrospray ion source. Immunoprecipitation followed by mass spectrometry was performed in three biological replicates. Samples were processed and RAW files generated were analyzed with MaxQuant (using standard parameters/) against the Uniprot *P. falciparum* reference proteome database. The protease used to generate peptides, i.e. enzyme specificity was set for trypsin/P (cleavage at the C terminus of “K/R: unless followed by “P”) along with maximum missed cleavages value of two.

### Quantitative RT-PCR

RNA isolation was carried out using TRIzol reagent (Biorad). 2 μg of DNAse free RNA was used for cDNA synthesis using ImProm-II Reverse transcription system (Promega), as per the manufacturer's recommendation. Random primers were used for the cDNA synthesis. Real time PCR was carried out using CFX96 Real Time PCR detection system (Biorad). 18S rRNA and tRNA synthetase were used as an internal control to normalize for variability across different samples. Quantification of the expression was done with the help of fluorescence readout of SYBR green dye incorporation into the amplifying targets (Biorad). Each experiment included technical replicates and was performed over three independent biological replicates. Primers details for the RT-qPCR are given in Supplementary Table S3.

### Chromatin immunoprecipitation

Infected RBCs were crosslinked using 1% formaldehyde (Thermo Scientific, 28908) for 10 min at RT. 150 mM glycine was added for quenching the cross-linking reaction. The samples were washed using 1X PBS (chilled) before proceeding with lysis. Sample homogenization was performed using swelling buffer (25 mM Tris pH 7.9, 1.5 mM MgCl2, 10 mM KCL, 0.1% NP 40, 1 mM DTT, 0.5 mM PMSF, 1 × PIC) followed by cell lysis in sonication buffer (10 mM Tris–HCl pH 7.5, 200 mM NaCl, 1% SDS, 4% NP-40,1 mM PMSF, 1X PIC). Sonication was performed using Covaris S220 to obtain the chromatin size of 200–400 bp. Pre-clearing was performed for 1 h at 4 °C using recombinant protein G conjugated sepharose beads with continuous gentle inverting. 30 μg purified chromatin was used per antibody and incubated for 12 h at 4 °C. Samples were then incubated with saturated Protein G Sepharose beads for 4 h at 4 °C. Bound chromatin was finally washed and eluted using ChIP elution buffer (1% SDS, 0.1 M sodium bicarbonate). Both IP sample and input were reverse crosslinked using 0.3 M NaCl overnight at 65 °C along with RNAse. Proteinase K treatment was performed at 42 °C for 1 h. Finally DNA was purified using phenol chloroform precipitation. Target sites identified from ChIP sequencing analysis were further validated by ChIP-qPCR using the Biorad SYBR Green Master Mix (Biorad). Primers details for the ChIP-qPCR are provided in Supplementary Table S4. Gene ontology was performed using PlasmoDB (www.plasmodb.org)^59^.

### ChIP-sequencing library preparation and sequencing

ChIP-sequencing libraries for all the samples were prepared from 5 to 10 ng of DNA using the NEBNext Ultra II DNA Library Prep kit. Chromatin immunoprecipitated, fragmented DNA samples were end repaired and adapters ligated. Size selection was performed using Agencourt AMPure XP beads (Beckman Coulter). Adapter ligated fragments were PCR amplified using indexing primers followed by purification using the Agencourt AMPure XP beads (Beckman Coulter). The library electropherograms were assessed using Agilent Bioanalyzer 2100 and Agilent DNA 1000 kit. The libraries were pooled in equimolar concentration and 50 bp reads were sequenced using Illumina HiSeq2500 (BENCOS Research Solutions Pvt. Ltd., Maharashtra). Gene which are bound to PfGCN5 under control and artemisinin treatment are provided in Supplementary Table S5.

### Data pre-processing and peak calling

ChIP-seq data were mapped to *Plasmodium falciparum 3D7* genome version 37 (http://plasmodb.org/plasmo/) using Bowtie2 with default parameters. The mapped reads were used for peak calling against an input control data, using the MACS2 peak calling software (https://github.com/macs3-project/MACS, default parameters)^60^. Peaks were annotated using Bedtools^61^. ChIP-seq signals were background subtracted using MACS2 bdgcmp tool and the significantly enriched peaks were visualized using Integrative Genomics Viewer (IGV).

### Average profile calculations

We extracted the tag density in a 5 kb window surrounding the gene body using the seqMINER tool which generates heatmap as well as the enrichment profiles of factors over gene bodies^62^. For average gene profiles, genes (± 5000 bp from binding site) were divided in 100 bins relative to the gene length. Moreover 10 equally sized (50 bp) bins were created on the 5′ and 3′ of the gene and ChIP-seq densities were collected for each dataset in each bin.

### Data source and analysis

Histone modification ChIP-seq data sets were downloaded from the database under the accession number GSE63369. seqMINER was used for generating scatter plots and average gene occupancy profiles^62^. Correlation analysis and box plot were generated using ‘R’ software (http://r-project.org/).

### Stress induction

Parasites were subjected to heat and therapeutic (artemisinin treatment) stresses for 6 h from late ring (~ 17 h) to early trophozoite (~ 23 h) stage. Double synchronization was carried out to achieve tight synchronization of parasite stages. Parasites were exposed to a) Heat stress (40 °C for 6 h) and b) Therapeutic stress (30 nM artemisinin for 6 h).

### RNA sequencing and data analysis

Parasites were harvested for RNA isolation after 6 h of stress induction. Total RNA was isolated using TRIzol reagent according to the protocol. DNAse treated RNA was used for cDNA synthesis. Quality of the RNA was verified using Agilent Bioanalyzer 2100. Three biological replicates were pooled together for performing RNA sequencing. The cDNA libraries were prepared for samples using Illumina TruSeq RNA library preparation kit. Transcriptome sequencing was performed using Illumina NextSeq 500 system (1 × 150 bp read length) at BioServe Biotechnologies (India) Pvt Ltd. Hyderabad in replicate. Quality control of the RNA-sequencing reads was performed using FASTQC and reads were trimmed based on the quality estimates. The quality verified reads were then mapped onto the reference genome (PlasmoDB_v37) using the HISAT2 software (New Tuxedo Suite). After verification of the mapping percentage, the alignment data (SAM format) was converted into its binary counterpart (BAM format) using SAMtools. The same step also sorts the aligned reads positionally according to their genomic coordinates, making them easier to process further. In order to quantify the reads mapped onto the genomic features (genes, exons, etc.), the htseq-count feature was used. The count data was then used to perform differential gene expression (DGE) analysis and statistical validation using the Deseq2 package in the R computational environment. MA plot is generated using ‘R’ software (http://r-project.org/).

### Ring stage survival assay (RSA)

In vitro RSA was performed according to the protocol described in Witkowski et al*.*^63^. Parasites were synchronized at early ring stage. Tightly synchronised 0–3 h rings were given 700 nM of artemisinin for 6 h. Drug was washed after 6 h with RPMI. Culture was then cultivated for 66 h. Parasites were then lysed and the parasite growth was calculated with the help of SYBR green I reagent which intercalates with the DNA and gives a fluorescent readout upon excitation. Parasite survival rate was calculated comparing the growth between drug treated and untreated control.

### Data access

ChIP-sequencing data for PfGCN5 as well as gene expression data (RNA sequencing) for different conditions are submitted to Sequence Read Archive (SRA) under ID SUB4919705 and SUB8323667.

### Ethics statement

This study does not involve human participants. Human RBCs used in this study were obtained from the KEM Blood Bank (Pune, India) as blood from anonymized donors. Approval to use this material for *P. falciparum *in vitro culture has been granted by the Institutional Biosafety Committee of Indian Institute of Science Education and Research Pune (BT/BS/17/582/2014-PID). The use of rabbits in this study for immunization (IISER/IAEC/2017-01/008) was reviewed and approved by Indian Institute of Science Education and Research (IISER)-Pune Animal House Facility (IISER: Reg No. 1496/GO/ReBi/S/11/CPCSEA). The approval is as per the guidelines issued by Committee for the Purpose of Control and Supervision of Experiments on Animals (CPCSEA), Govt. of India.

## Supplementary Information

Supplementary Information 1.

Supplementary Information 2.

Supplementary Information 3.

Supplementary Information 4.

## Abbreviations

RBC: Red blood cells
HPI: Hours post invasion
ROS: Reactive oxygen species
ChIP-seq: Chromatin immunoprecipitation coupled high-throughput sequencing
HAT: Histone acetyltransferase
RT-qPCR: Quantitative real-time PCR
GO: Gene ontology
RSA: Ring survival assay
