# Supplementary material for: Histone acetyltransferase PfGCN5 regulates stress responsive and artemisinin resistance related genes in Plasmodium falciparum
Source: Sci Rep. 2021 Jan 13;11:852. doi: 10.1038/s41598-020-79539-w (PMC7806804; doi:10.1038/s41598-020-79539-w)
Supplement: Supplementary file 4 — Supplementary Information 4. [file 41598_2020_79539_MOESM4_ESM.docx]

**Histone acetyltransferase PfGCN5 regulates stress responsive and artemisinin resistance related genes in *Plasmodium falciparum***

Mukul Rawat^1^, Abhishek Kanyal^1^, Aishwarya Sahasrabudhe^1^, Shruthi Sridhar Vembar^2^, Jose-Juan Lopez-Rubio^3^ and Krishanpal Karmodiya^1*^

^1^Biology Department, Indian Institute of Science Education and Research, Dr. Homi Bhabha Road, Pashan, Pune 411 008, India;

^2^Institute for Bioinformatics and Applied Biotechnology, Bengaluru, Karnataka, India;

^3^Laboratory of Pathogen-Host Interactions (LPHI), UMR5235, CNRS, INSERM, Montpellier University, Montpellier, France.

**Running title:** PfGCN5 upregulates stress responsive genes during stress conditions

*To whom correspondence should be addressed: Krishanpal Karmodiya: Department of Biology, Indian Institute of Science Education and Research, Dr. Homi Bhabha Road, Pashan, Pune 411 008, India; [krish@iiserpune.ac.in](mailto:krish@iiserpune.ac.in); Tel. (+91 (20) 2590 8195)

**Supplementary Figures**

**
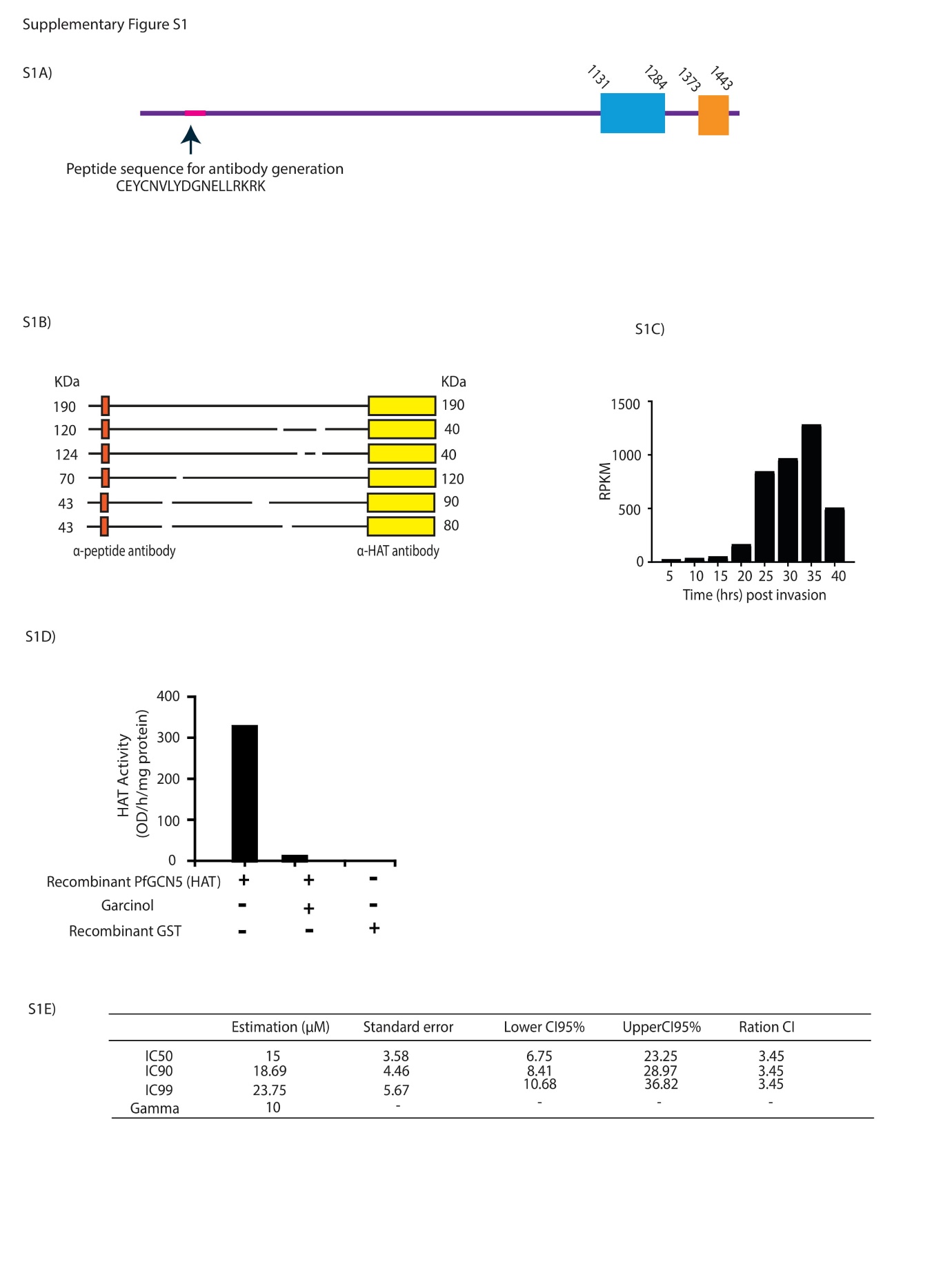
**

**Supplementary Figure S1: *Pf*GCN5 specific antibody generation.** (S1A) Schematic diagram showing the domain organization of PfGCN5. Histone acetyltransferase
(HAT) domain and bromodomain (represented in blue and orange colour, respectively) are present at C terminal end. PfGCN5 peptide from N-terminal region of the protein was commercially synthesized for raising antibody. (S1B) Schematic to explain the possible bands generated using the *in silico* proteolytic analysis tool, PROSPER (<https://prosper.erc.monash.edu.au/queue.pl>). Various bands observed with PfGCN5 N-terminal peptide antibody and C-terminal HAT antibody are shown. (S1C) Dynamics of PfGCN5 transcript expression during different stages of intraerythrocytic life cycle of *P. falciparum.* Expression profile suggests the low expression of PfGCN5 during the ring stages and a sudden burst of PfGCN5 mRNA expression during early trophozoite stage. (S1D) Inhibition of histone acetylation activity of purified recombinant HAT domain of PfGCN5. 10 µM of Garcinol inhibits PfGCN5 HAT activity completely. (S1E) IC50 calculation of garcinol using dose response assay carried out over a period of 48 hours. The growth inhibition was measured using the SYBR green dye.


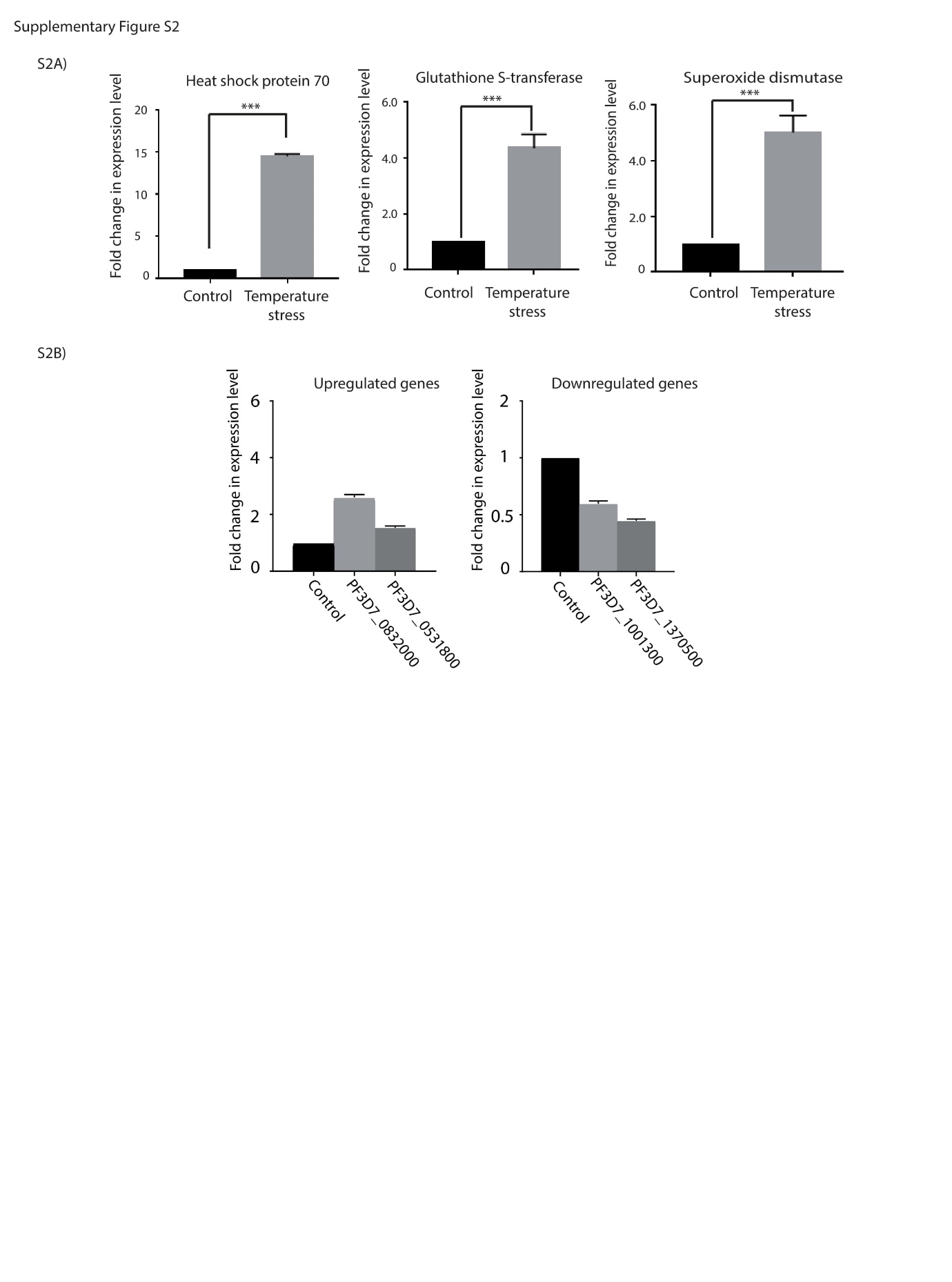


**Supplementary Figure S2: Differential gene expression during stress conditions.** (S2A) Different marker genes were found to be deregulated during stress conditions. Temperature stress results in up regulation of HSP70. Similarly artemisinin (ART) treatment results in the increase in expression of Glutathione S-transferase and Superoxide dismutase which indicates the presence of ROS in parasites due to artemisinin treatment. Up regulation of these genes is indicative of the fact that stress is induced in the parasite upon artemisinin treatment and increase in temperature. (S2B) RT-qPCR validation of the genes which are deregulated during stress conditions, identified through RNA sequencing.


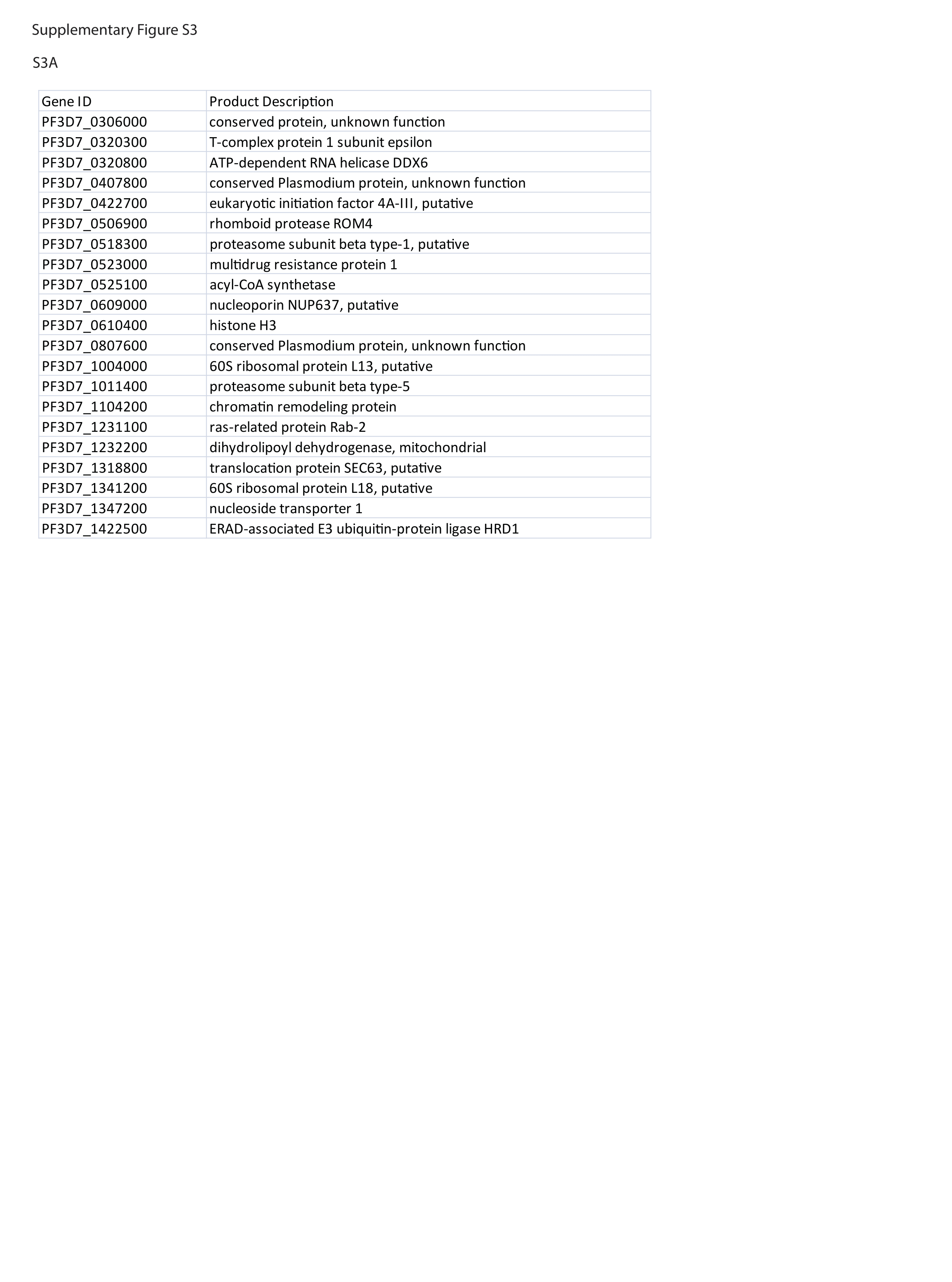


**Supplementary Figure S3: Gene upregulated found to be upregulated in artemisinin treatment and bound by PfGCN5 under artemisinin treatment.** (S3A) Table listing the overlapping genes which were bound by PfGCN5 under artemisinin treatment (30 nM) in our study and were found to be upregulated in a previously reported proteomics study under artemisinin treatment (35 nM).

**Supporting information Table legends**

**Supplementary Table S1: Immunoprecipitation performed with α-peptide antibody using IgG as control.** List of protein identified in the mass spectrometry analysis for the α-peptide antibody and IgG antibody. Out of 223 proteins identified with PfGCN5 pull down 87 were present in IgG pull down also. Only 136 proteins were specific to PfGCN5 pulldown which contain PfGCN5 as well its known interacting partner PfADA2.

**Supplementary Table S2: Genes deregulated during stress conditions.** RNA sequencing was performed during stress conditions (Artemisinin treatment and Temperature stress) to identify the genes deregulated. List of genes along with their tag count is listed in **Table S2**. Gene ontology terms of gene upregulated under treatment are also included in the same file.

**Supplementary Table S3: Primers used in the study.** Sequences of the RT-qPCR primers used in the study.

| Gene | Forward Primer | Reverse Primer |
| --- | --- | --- |
| GCN5 | TGCTGATAATAAAGGGGCTGC | AATGGCCATGCAGACTGTTG |
| GCN5 | GGAATCGGGCATATCGATTAAC | CCGCATCTCTGTGCAAATATC |
| Glutathione S transferase | ATGCAAGGGGTAAAGCTGAA | TCAACAAAAGCATCACCGTTT |
| HSP70 | TGCTGATAACCAACCAGGTG | TTTCTTGGTGCAGGTGGAAT |
| Superoxide dismutase | CAAGGTGGAGGAATGCCATA | CCACCCACTTCCAAAATGAC |
| PF3D7_1370500 | GGATAGCTGTTCTGACCACATTT | GGTACTTAACCCTCCCCGTAG |
| PF3D7_0832000 | TGGTAATGTTGCTGCTGCTT | TGGGGCTGAAAAAGCACTTA |
| PF3D7_0531800 | CGATGGATGTCTTGGTTCCT | AGTACCACTATTCCCCGAAGG |
| PF3D7_1001300 | AAGAAATTCCACCAAAACGTG | TCACAATCATATGGGTTATTCTT |
| tRNA synthetase | TGGAACAATGGTAGCTGCACAAA | ATGGGCGCAATTTTTCAGGA |
| 18s RNA | GCTGACTACGTCCCTGCCC | ACAATTCATCATATCTTTCAATCGGTA |
| PF3D7_0206800 | TAATGGTGCAGATGCTGAGG | TGGATTTGTTTCGGCATTTT |
| PF3D7_0200100 | ATGTGCGCTACAAGAAGCTG | TTGATCTCCCCATTCAGTCA |
| PF3D7_0420900 | AGAGGGTTATGGGAATGCAG | GCATTCTTTGGCAATTCCTT |
| PF3D7_0304600 | ATGCAAACCCAAATGCAAAC | GGTTTGGGTCATTTGGCATA |
| PF3D7_0515300 | CGTGTTTGGGCGATAAACTGA | CCTAACGAGGAAACGCCTGA |
| TCP1 beta (PF3D7_0306800) | TGGATTTCGTATGGCATTAGC | GCTAGCTCGGCAAAATGTTC |
| BIP (PF3D7_0917900) | TGAGGGACCCGTTATTGGTA | TGCCTCACCAACTTTCCTTT |

**Supplementary Table S4: Primers used in the study.** Sequences of the ChIP-qPCR primers used in this study.

| Genes | Forward primer | Reverse Primer |
| --- | --- | --- |
| Pf3D7_0506800 | TGTTGGGTTAAACATACCACCA | GGAGGCATCCATATGCTGTT |
| Pf3D7_0202000 | AACTGTTCCCTGGGGTTGTT | TCAACACCAACACCAAGCTC |
| Pf3D7_1410600 | TGTGGCTCATGGAAAATCAA | TTCAGGTGGTAAGCAATCAGG |
| Pf3D7_1022400 | TGGGTCATCTGGAAAGTGTG | CATGGACTAGCCAATCAAGAA |
| Pf3D7_0807500 | GTTTTTGTGCGGGATATCGT | GCTTCAATTGCCAAAATGGT |

**Supplementary Table S5: Genomic sites and genes bound by PfGCN5 identified using ChIP sequencing.** ChIP sequencing of PfGCN5 using the α-peptide antibody was performed during early trophozoites. The sites identified to be bound by PfGCN5 are listed in the table according to their decreasing fold enrichment. Genes identified to be bound by PfGCN5 are listed in the same table.
